# Ten years of a gender identity service for children and adolescents in Germany: demographic and clinical patterns

**DOI:** 10.1007/s00787-026-03080-w

**Published:** 2026-06-08

**Authors:** Lena Herrmann, Claus Barkmann, Saskia Fahrenkrug, Sarah Hohmann, Carola Bindt, Clara Lammers, Inga Becker-Hebly

**Affiliations:** Department of Child and Adolescent Psychiatry, Psychotherapy and Psychosomatics, University Medical Centre Hamburg-Eppendorf, Martinistr. 52, 20246 Hamburg, Germany

**Keywords:** Transgender, Gender diverse, Gender dysphoria, Sex ratio, Clinical characteristics, Psychological functioning

## Abstract

Key discussions in current research on transgender and gender diverse children and adolescents include rising referral rates of adolescents assigned female at birth (AFAB) compared to adolescents assigned male at birth (AMAB) to specialized gender identity services and shifts in demographic and clinical characteristics of clients. The present study aimed to investigate two things: (1) a potential linear increase in the proportion of AFAB (vs. AMAB) referrals over time; and (2) to identify distinct clinical patterns based on time, sex assigned at birth, and clinical characteristics, such as psychological functioning and onset age. A clinical sample of children and adolescents (n = 779) attending a specialized gender identity service in Germany (Hamburg GIS) over ten years, between September 2013 and September 2023, was assessed. Cross-sectional information provided by parents and clinicians included the year of initial visit, sex assigned at birth, psychological functioning (Child Behavior Checklist), and onset age of gender dysphoria (pre-pubertal vs. post-pubertal onset of gender dysphoria based on DSM-5 criteria in childhood and adolescence). A logistic regression analysis was used to test for a linear time trend in sex assigned at birth. A latent class analysis (LCA) was performed on the year of initial visit, sex assigned at birth, and clinical characteristics (psychological functioning and onset age) in order to identify potential clinical patterns. Even though the sample consisted of almost four times more AFAB compared to AMAB referrals, there was no significant linear increase in the proportion of AFAB children and adolescents over time. LCA revealed two distinct cohorts: an earlier one (mean year of initial visit: mid-2016) and a more recent one (mean year of initial visit: around 2021). These cohorts differed significantly with regard to all demographic and clinical characteristics. The more recent (2021) cohort included a higher proportion of AFAB individuals, less problematic psychological functioning in the clinical range, and more cases of post-pubertal onset (late onset) compared to childhood (early onset) gender dysphoria than the earlier cohort (2016). In conclusion, there was no evidence of a linear increase in the proportion of AFAB children and adolescents over time. However, two distinct clinical patterns were identified, one earlier cohort (referred around 2016) and one more recent cohort (referred around 2021), and these differed in various demographic and clinical characteristics. Long-term research is required to determine how these patterns fit with particular clinical needs.

**Supplementary Information:**

The online version contains supplementary material available at https://doi.org/10.1007/s00787-026-03080-w.

## Introduction

Over the past two decades, specialized services for transgender and gender diverse (TGD) children and adolescents in the Western hemisphere have reported a marked rise in referrals [1–10]. Similarly, the prevalence of the diagnosis gender dysphoria (GD) in the general population has increased significantly over the last ten years [11, 12]. In addition, the diversity of clinical presentations among youth attending gender identity services is being more widely recognized: This includes variations in gender identity (e.g., non-binary identities), co-occurring mental health diagnoses (e.g., anxiety, depression, and autism), and developmental pathways (e.g., "late", adolescent, or post-pubertal onset of gender dysphoria) [13–17]. These developments have sparked controversial debates about the applicability of current treatment protocols to more recent and diverse referrals [13, 18], influenced restrictions on transition-related medical care for TGD youth in the United States [19], and prompted a change in healthcare practices in Europe [20]. Further information on potential changes to the demographic and clinical characteristics of referrals is urgently needed to ensure that this vulnerable group receives safe and beneficial treatment in each individual case.

The term TGD is used to describe individuals whose gender does not (fully) align with their sex assigned at birth [21]. If this gender incongruence is marked, lasts for at least six months, and is accompanied by significant clinical distress, the diagnosis of GD is applicable [22]. Population estimates of TGD individuals vary considerably across studies. While the prevalence of GD diagnoses in health system-based studies is estimated to be around 0.02 to 0.08% [11, 23], approximately 1 to 3% of children and adolescents identify as TGD in survey-based studies [24–26]. Over the past decade, the estimated prevalence of GD has increased significantly, while the mean age of first presentation at a clinic has been decreasing [11, 12].

At the same time, there have been shifts in the demographic and clinical characteristics among patients seeking care. One of the most prominent discussed recent developments is the shift in the sex ratio of adolescent referrals to specialized gender identity services in North America, Israel, and Europe in favor of assigned female at birth (AFAB) individuals over the past two decades [1–3, 8, 27, 28]. For example, the sex ratio of referrals to a Dutch gender identity service now favors AFAB adolescents, whereas it was relatively balanced until 2012 [29]. Although there is currently no comparable clinical study from Germany, where the present study took place, the sex ratio among young German applicants for legal name and gender change shifted substantially towards AFAB youth between 2005 and 2019 [30]. Since the start of data collection at our respective center in Hamburg almost 13 years ago, the sex ratio of referrals has consistently favored AFAB adolescents [16, 31–33].

For children attending specialized gender identity services, however, the evidence is more inconclusive. While some studies suggest significant changes in the sex ratio, most still report either a continued overrepresentation of assigned male at birth (AMAB) referrals or a relatively balanced ratio [3, 27, 29, 34, 35]. Most studies report no significant change in the sex ratio among children [28, 36]. While the underlying reasons for these differences between child and adolescent samples are unclear, discrepancies in the study periods likely contribute to the observed discrepancies. Furthermore, the differences may reflect variations in care and treatment pathways, as well as cultural differences.

In addition to the sex ratio, a few studies have focused on time trends in other characteristics, including clinical factors such as psychological functioning. Concerning psychological functioning, one study from the UK observed significantly more diagnoses of mood and anxiety disorders in AFAB (but not AMAB) youth in a more recent cohort (from 2015) than in an earlier one (from 2012) [37]. In contrast, no significant changes in psychological functioning over time were found in another study from Canada [38], while one Dutch study even reported a significant improvement in psychological functioning as measured by the Child Behavior Checklist and Youth Self-Report over time [8].

As a theoretical explanation for changes in the clinical presentation of youth with GD, some authors have suggested that the developmental pathways of more "recent" clinical referrals may differ from those of "earlier" referrals [13, 16, 42]. Studies, such as our own published by Fahrenkrug et al. (2025), have focused on different developmental pathways of current referrals based on age at initial visit or GD onset age [16, 39–41]. Across these studies, a higher age at the initial visit or a post-pubertal (late) onset (i.e., GD onset after puberty) was significantly associated with reporting worse psychological functioning or more mental health problems [16, 40, 41]. For instance, a post-pubertal onset was significantly associated with a higher prevalence of internalizing problems among TGD adolescents in a recent study undertaken by our team [16]. Although the proportion of post-pubertal-onset cases compared to pre-pubertal-onset cases (i.e., GD onset before puberty), especially in AFAB adolescents, has probably increased over time [13, 16, 42], it remains unclear whether the developmental pathways of more recent referrals differ from those of earlier referrals (as this has not yet been investigated). 

## Current study

In summary, several studies suggest a temporal shift in the demographic and clinical characteristics of youth attending specialized gender identity services. However, except for the change in sex ratio, the evidence concerning this temporal shift is ambiguous (i.e., unclear direction of change) or lacking (in the case of onset age). Thus, it remains unclear whether current referral patterns differ from those observed in earlier cohorts, which would suggest that clinical patterns may have changed over time. Therefore, the present study examined a potential change in the sex ratio and the presence of distinct clinical patterns among children and adolescents attending a gender identity service in northern Germany. The following research questions were addressed:

1. Linear time trend: Has the sex ratio in referrals of children and adolescents attending a specialized gender identity service in Germany changed over the past 10 years (2013–2023), and if so, how?
2. Clinical patterns: Can different clinical patterns be identified based on time of initial visit/referral, sex assigned at birth, and other clinical characteristics (such as psychological functioning and onset age)?

We hypothesized that there would be a linear increase in the proportion of AFAB referrals compared to AMAB referrals. There was no hypothesis for the second research question, so it was evaluated in an exploratory way.

## Methods

### Study design and population

The present study used a cross-sectional design, where each participant was surveyed only once, and assessed children and adolescents who had attended the Hamburg Gender Identity Service for children and adolescents (Hamburg GIS) between September 2013 and September 2023. The Hamburg GIS is located at the University Medical Center Hamburg-Eppendorf in Germany, and provides specialized diagnostics, counseling, and treatment for TGD children and adolescents and those with questions regarding their gender or sexual identity. The clinical protocol at the Hamburg GIS applies a developmental perspective on GD and generally follows international treatment guidelines, but with a strong focus on individualized care for TGD children and adolescents. After a comprehensive diagnostic evaluation over multiple sessions, further psychological support as well as gender-affirming medical interventions may be offered if adolescents meet certain criteria, such as a sustained wishing for treatment, persistent GD, absence of severe mental health issues/psychiatric diagnoses that could interfere with the clarity of gender identity development or decision making, adequate psychosocial support during treatment, and sufficient understanding of the potential consequences of gender-affirming medical interventions (for more information see [33, 43]).

**Fig. 1:**
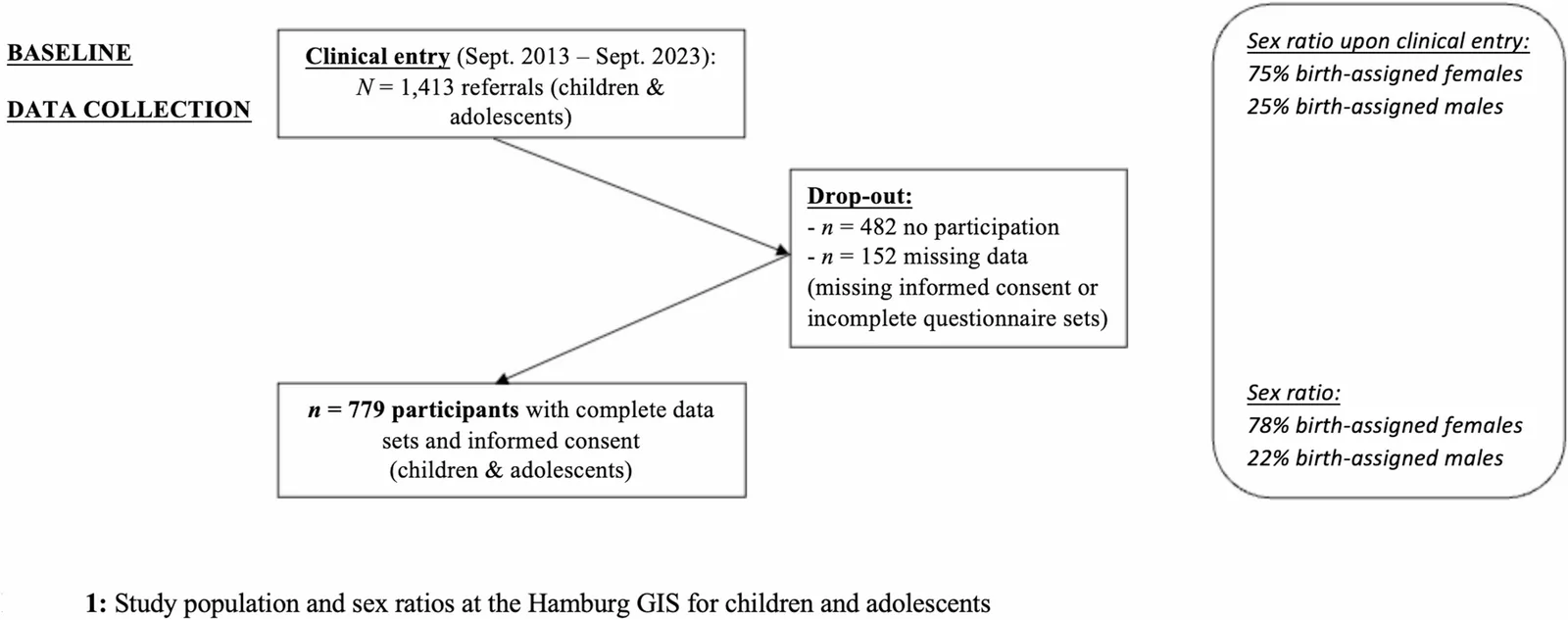
Study population and sex ratios at the Hamburg GIS for children and adolescents

Since 2013, all families have been invited to participate in the questionnaire-based study at their first appointment at the Hamburg GIS, before any form of treatment or counseling. By the end of the present study period in 2023, a comprehensive set of questionnaires had been administered to approximately 1,400 families attending the service (Fig. 1). Previous publications from this service have examined various aspects of the referred population, including clinical characteristics [31], co-occurrence with autism [44], onset age [16], (non-binary) gender identities [14], mental health [32, 45], quality of life [46], body image [47], treatment pathways [33], treatment satisfaction [48], and psychosocial health outcomes following two years of gender-affirming medical treatment [43]. However, none of our studies to date have investigated the emergence of different clinical patterns over the entire observed time period of the research project. For the present study, parents' or carer-givers' reports instead of self-reports were used in order to be able to include both children (up to 10 years old; who did not provide self-report) and adolescents (11 years and older). Additionally, clinicians filled out a diagnostic questionnaire for each participant.

Participation in the questionnaire-based study was voluntary. Ethical approval was granted by the local ethics committee (12/2019-PTK-HH). Written informed consent was obtained from all participating adolescents and their parents/caregivers.

### Study population

We analyzed data from all families who participated in the questionnaire-based study during the study period. Of the *N* = 1,413 referrals between September 2013 and September 2023, *n* = 779 participants provided complete data sets (comprising complete questionnaire sets and informed consent). We chose to include all participants regardless of their GD diagnosis or history of gender-affirming treatment. This decision was made to ensure a comprehensive understanding of this diverse group. For a detailed overview of the study population, please refer to Fig. 1.

We checked for selection bias based on sex assigned at birth and age at initial visit and found no significant differences between all referrals (*N* = 1,413) and study participants (*n* = 779). Neither age (*t*(2190) = − 1.24, *p* = .214, *d* = 0.06) nor sex assigned at birth (χ^2^ (1, *N* = 2,192) = 3.09, *p* = .079, OR = 1.21) differed significantly between the two groups. An overview of the demographic characteristics of the entire referral sample can be found in Table A1 of the Appendix.

### Variables and instruments

#### Year of initial visit

The year of initial visit, i.e., the calendar year in which the child/adolescent first presented to the Hamburg GIS, was centered around the first study year 2013 (by subtracting 2013 from all values) to facilitate interpretation of the regression analysis.

#### Demographic characteristics

The following demographic characteristics of the children and adolescents were considered in the analyses: sex assigned at birth and age at initial visit. Based on the age at initial visit, two age groups were differentiated: children (up to 10 years old) and adolescents (11 years or older).

#### Clinical characteristics

The German version of the Child Behavior Checklist (CBCL) [49, 50] was used to measure psychological functioning in children and adolescents. The CBCL was completed by the parents/caregivers of the children and adolescents. CBCL items were rated on a 3-point scale ranging from 0 (“not true”) to 2 (“very true or often true”) and referred to the past six months. *T* scores were calculated for the total problems score based on the German population-based, age- and sex-specific norms provided by Döpfner et al. [50] for the German version of the CBCL from 1991 [50], with participants’ sex assigned at birth used as the reference, as it has been done before in similar studies [17, 52]. Afterwards, T scores were used to build clinical range scores (> 90th percentile; *T* scores > 63).

The following items were excluded from the calculation of the total problems score: asthma (Item 2), allergies (Item 4), and cross-gender identification (Item 5 and Item 110). The latter items were still included in the 1991 version of the CBCL used in the study [50]. As suggested by similar studies [51, 52], Item 84 and Item 85 were set to zero if the free-text answers were gender-related, as these could result in artificial inflation. In the present study, the reliability of the CBCL total problems score was deemed excellent, with Cronbach’s α = .94.

For onset age, specialized clinicians working with the children and adolescents and their families assessed the criteria for GD during the diagnostic sessions with a study-specific checklist based on the DSM-5 [22]. Three categories were built: “pre-pubertal (early) onset” (criteria for GD already fulfilled in childhood/before the onset of puberty), “post-pubertal (late) onset” (criteria for GD fulfilled in adolescents/after the onset of puberty but not in childhood), and “no GD diagnosis” or “missing information” (cf. [16]).

### Statistical analyses

Descriptive analyses included calculating frequencies, means, and standard deviations. Exploratory binomial tests were conducted to assess whether the observed sex ratios differed significantly from an equal distribution.

To test our first hypothesis (linear increase in AFAB referrals over time), we conducted a multiple logistic regression analysis in a blockwise manner. In the first step, year of initial visit was the only predictor, with sex assigned at birth as the binary outcome. In the second step, age group, clinical ranges of the CBCL total problems score, and onset age (dummy coded, with pre-pubertal onset of GD as the reference category) were added as additional predictors.

Furthermore, to investigate the second research question concerning the presence of distinct clinical patterns, an exploratory latent class analysis (LCA) was conducted. As our approach was exploratory, LCA was chosen for its ability to identify patterns in complex, multivariate data. Unlike regression-based methods, which require predefined groups or test specific relationships between variables, LCA detects unobserved patterns (latent classes) within a population based on their patterns of responses to a set of observed categorical variables (indicator variables). Individuals with similar characteristics are grouped, even if these characteristics are not directly measured [53]. The analysis included year of initial visit, sex assigned at birth, clinical ranges of the CBCL total problems score, and onset age as indicator variables. The Bayesian Information Criterion (BIC), the most reliable traditionally used information criterion for model selection [54], the sample-size adjusted BIC (aBIC), entropy values, and the Lo-Mendell-Rubin adjusted likelihood ratio test (LMR-LRT) were employed to determine the optimal number of classes. Specifically, non-significant LMR-LRT *p*-values suggested that adding an extra class did not significantly improve the model compared to one with fewer classes. Lower BIC values indicated better model fit, while entropy values closer to one reflected more distinctly defined classes. Ultimately, the decision on the optimal number of classes was based on these statistical fit indices combined with the principle of parsimony.

Additional exploratory analyses were conducted to examine whether using the CBCL internalizing and externalizing scales, rather than the CBCL total problems score altered the results of the regression analysis and LCA, and whether using cross-sex norms instead of birth-assigned sex norms for the total problems score led to different results. First, the multiple regression analysis testing a potential linear increase in AFAB individuals over time was repeated three times as described above, with the internalizing scale, externalizing scale, and total problems score based on the cross-sex norms entered as predictors in three distinct models in place of the total problems score. Second, three separate LCAs were conducted as described above, using the clinical ranges of the CBCL internalizing and externalizing scales, as well as the total problems score based on the cross-sex norms as categorical indicators, instead of the total problems score based on birth-assigned-sex norms.

Single missing values were imputed with the expectation maximization algorithm [55], consistent with previous studies [14, 16, 32, 45]. Missingness ranged from 0% to 18% across items and participants, with the higher percentages observed for a few CBCL items. Statistical analyses were performed using SPSS 29 and MPlus version 8.

## Results

### Demographic and clinical characteristics

Demographic and clinical characteristics of the study population (*n* = 779) are presented in Table 1. Over the 10-year period, AMAB children and adolescents accounted for one-fifth of the study population, whereas AFAB children and adolescents made up four-fifths, corresponding to a sex ratio of 3.7:1.0 (F: M). An exploratory binomial test indicated that this distribution significantly differed from an expected 1:1 ratio (*p* < .001). Compared to the entire study population, the sex ratio was more balanced among children (1.0:1.6, F: M), with a slight but non-significant predominance of AMAB individuals (61% AMAB vs. 39% AFAB; binomial test, *p* = .056). In contrast, among adolescents, the sex ratio was more unbalanced (5.1:1.0, F: M), with a strong and significant predominance of AFAB individuals (16% AMAB vs. 84% AFAB; binomial test, *p* < .001).

**Table 1:** Association between year of initial visit and sex assigned at birth in the study population (*n* = 779)

	*n*	%
Sex assigned at birth		
Assigned male at birth	167	21.4
Assigned female at birth	612	78.6
Age group		
Children (up to 10 years old)	89	11.4
Adolescents (11 years or older)	690	88.6
CBCL total problems score	305	39.2
Non-clinical range (*T* scores ≤ 63)	474	60.8
Clinical range (> 90th percentile; *T* scores > 63)	305	39.2
Onset age		
Pre-pubertal onset of GD	370	47.5
Post-pubertal onset of GD	334	42.9
No GD diagnosis or missing information	75	9.6

CBCL, Child Behavior Checklist; GD, gender dysphoria

At the time of their first presentation to the Hamburg GIS, nearly 90% of participants were already in adolescence, defined as 11 years or older. The overall mean age at initial visit was *M* = 14.59 years (*SD* = 2.87), with the data being approximately normally distributed.

With regard to parent-reported psychological functioning, 40% of the participants scored in the clinical range on the CBCL total problems score (above the 90th percentile, with *T* scores above 63).

Around half of the study population presented with a pre-pubertal (early) onset of GD, meaning it had set in before puberty. By contrast, 43% experienced a post-pubertal (late) onset, i.e., GD had set in after puberty had begun. The remaining 10% of children and adolescents had not been diagnosed with GD by the end of the evaluation process, or information was missing.

### Sex ratio over time

**Fig. 2:**
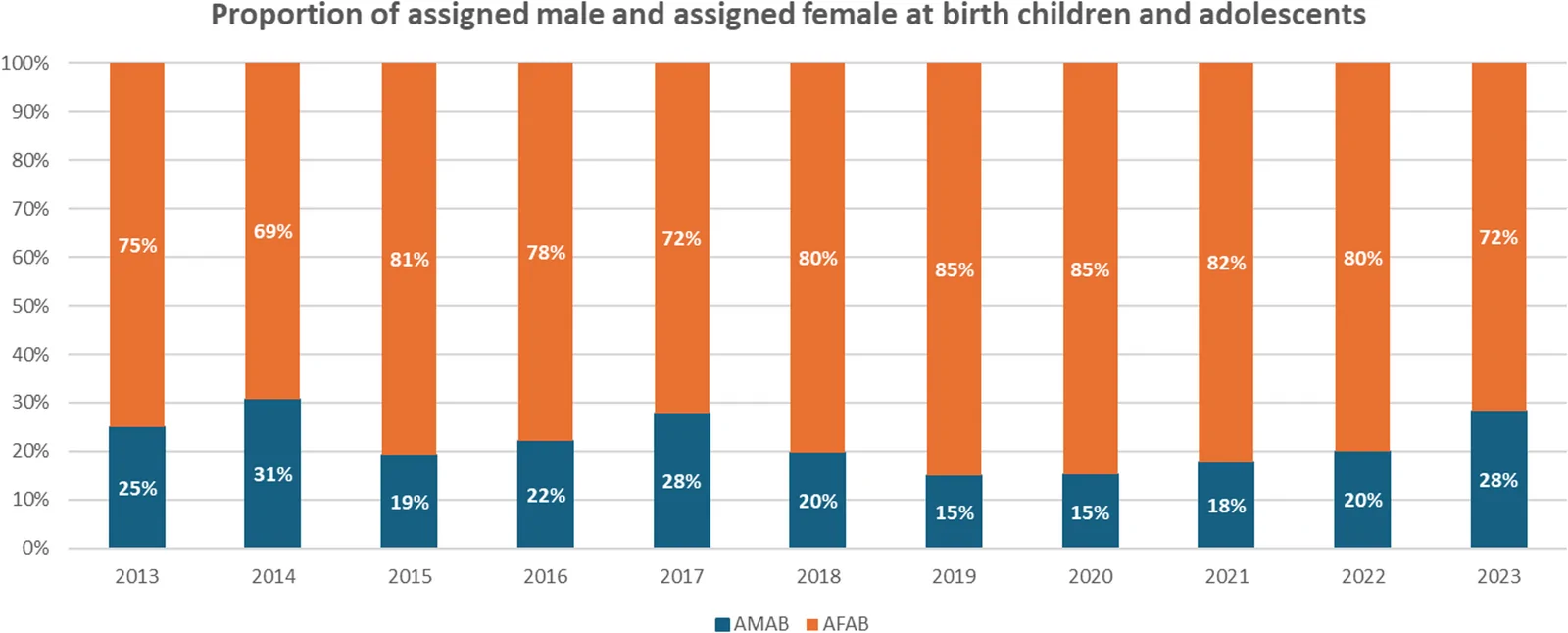
Proportion of assigned male at birth (AMAB) and assigned female at birth (AFAB) children and adolescents attending the Hamburg Gender Identity Service for children and adolescents between 2013 and 2023

Table 2 summarizes the results of the prediction of a potential change in the sex ratio over time. Contrary to our hypothesis, year of initial visit was not significantly associated with sex assigned at birth. This result indicates no evidence of a linear time trend in the proportion of AFAB relative to AMAB children and adolescents over the 10 years evaluated. As can be seen in Fig. 2, the proportion of AFAB children and adolescents compared to AMAB ones fluctuated over the ten-year period, with AFAB individuals making up between 69% and 85% of the study population.

**Table 2:** Association between year of initial visit and sex assigned at birth in the study population (n = 779)

	B	SE	OR	95% CI for OR	*p*
Intercept	-82.28	73.15	0.00		.261
Year of initial visit (centered)	0.04	0.04	1.04	[0.97, 1.12]	.263
Age group (0 = child, 1 = adolescent)	2.05	0.26	7.76	[4.62, 13.01]	< .001
CBCL clinical range of the total problems score (0 = non-clinical, 1 = clinical)	0.43	0.20	1.54	[1.04, 2.28]	.031
Post-pubertal onset of GD (= 1, pre-pubertal = 0)	−0.13	0.22	0.88	[0.57, 1.37]	.578
No GD or missing information (= 1, pre-pubertal = 0)	−0.51	0.31	0.60	[0.33, 1.09]	.095

Multiple logistic regression analysis, year of assessment centered on the year 2013, model fit: *χ*^2^(5) = 82.60, *p* < .001, *R*^*2*^ (Nagelkerke) = .16

GD, gender dysphoria

Significant predictors of sex assigned at birth in the multiple logistic regression analysis were age group and the CBCL clinical range scores. The odds of being AFAB were approximately eight times higher for adolescents compared to children, and 1.5 times higher for individuals scoring in the clinical range on the CBCL total problems score compared to those who did not. The model explained 16% of the variance in sex assigned at birth, which was entirely attributable to the additional predictors added in the second step of the analysis.

### Analysis of latent classes

Tables 3, 4 and 5 show the results of the LCA used to explore distinct clinical patterns. Based on the model fit indices, a 2-class model was selected as the best-fitting solution. Although the 3- and 4-class models had slightly lower sample-size adjusted BIC values, the Lo-Mendell-Rubin adjusted likelihood ratio test (LMR-LRT) was significant for the 2-class versus 1-class comparison but not for the 3-class versus 2-class or 4-class versus 3-class comparison. Entropy values indicated acceptable classification precision for the selected 2-class model (see Table 2). Classification quality of the 2-class model was accurate, with high average posterior probabilities for the most likely class membership. Individuals assigned to Class 1 had a 90% probability of belonging to that class. Whereas those assigned to Class 2 had a 93% probability, indicating a good separation between the two classes (see Table 4).

**Table 3:** Model fit for analyses of latent classes

Classes	LL	BIC	aBIC	Npar	Entropy	LMR-LRT	*p*
**2 (vs. 1)**	**-3463.97**	**7007.84**	**6969.73**	**12**	**.71**	**152.48**	**< .001**
3 (vs. 2)	-3450.28	7020.40	6963.24	18	.65	26.72	.215
4 (vs. 3)	-3432.65	7025.10	6948.89	24	.72	34.39	.058

(a)*BIC,* (sample-size adjusted) Bayesian information criterion; *LL,* Loglikelihood H0 value; *LMR-LRT,* Lo-Mendell-Rubin adjusted likelihood ratio test; Npar, number of free parameters

**Table 4:** Average latent class probabilities for most likely latent class membership (row) by latent class (column)

	1	2
1	.902	.098
2	.068	.932

The two emerging classes were labeled according to the year of initial visit, as this variable showed a substantial difference between both classes and aligned with the primary focus of the study. Class 1 (*n* = 409, 53%) represented an **earlier cohort** of children and adolescents who, on average, first attended the Hamburg GIS in mid-2016. Class 2 (*n* = 370, 47%) comprised a significantly **more recent cohort**, with an average year of initial visit of around 2021. The earlier cohort and the more recent cohort were distinct from each other, differing significantly across all demographic and clinical characteristics (see Table 5).

**Table 5:** Conditional response probabilities for categorical indicators and means (standard errors) for continuous indicators by latent class

	Class 1: earlier cohort (*n* = 409, 53%)	Class 2: more recent cohort (*n* = 370, 47%)	OR	*p*
	Sex assigned at birth			
Assigned male at birth	.24	.19		
Assigned female at birth	.76	.81	4.86	< .001
CBCL total problems score				
Non-clinical range (*T* scores ≤ 63)	.59	.63		
Clinical range (*T* scores > 63)	.41	.37	5.89	< .001
Onset age				
Pre-pubertal onset of GD	**.64**	.30		
Post-pubertal onset of GD	.26	**.62**	5.32	< .001
No GD diagnosis or missing information	.10	.09	3.33	.001
Year of assessment	2016.50 (0.11)	2020.88 (0.13)		< .001

*CBCL *Child behavior checklist, *GD *gender dysphoria

First, sex assigned at birth was significantly associated with class membership. Although both classes predominantly comprised AFAB individuals, the proportion was even higher in the more recent cohort (81%) than in the earlier one (76%).

Second, there was a significant association between psychological functioning and class membership: individuals in the more recent cohort were less likely to score in the clinical range of the CBCL total problems score (37%) compared to those in the earlier cohort (41%). Third, onset age was significantly associated with class membership. The more recent cohort was characterized mainly by post-pubertal onset cases (62%), whereas the earlier cohort showed a clear predominance of pre-pubertal onset cases (64%).

### Post-hoc data analysis

For further exploratory purposes, we conducted a multiple logistic regression analysis to examine the association between year of initial visit and sex assigned at birth within the full sample of referrals (*N* = 1,413). Consistent with the findings in the participant sample, there was no significant association between year of initial visit and sex assigned at birth. Detailed results of these exploratory analyses are presented in Table A2 and Figure A1 in the Appendix.

Further exploratory analyses of the sex ratio over time were performed using the CBCL internalizing and externalizing scale instead of the total problems score as the predictor, and using cross-sex norms instead of birth-assigned sex norms for the total problems score. Consistent with the main analyses, the year of initial visit was not significantly associated with sex assigned at birth in the multiple logistic regression models, indicating no change in the sex ratio over time when internalizing and externalizing problems as well as the total problems score based on cross-sex norms were included (in place of the total problems score based on the birth-assigned sex norms). Detailed results for these additional analyses are provided in Tables A3 to A6 in the Appendix.

Additional exploratory LCAs were conducted using internalizing and externalizing problems as categorical indicators instead of the total problems score and using cross-sex norms instead of birth-assigned sex norms for the total problems score. The results closely mirrored those of the main analyses: Two distinct classes emerged, showing significant differences across all demographic and clinical characteristics. The more recent cohort included a significantly higher proportion of AFAB individuals, more cases with post-pubertal onset, and scored less often in the clinical range of the externalizing scale and the total problems score (based on the cross-sex norms) than the earlier cohort. However, in contrast to the main analyses, individuals in the more recent cohort were significantly more likely to score in the clinical range on the internalizing scale than those in the earlier cohort. Detailed results for these additional analyses are presented in Tables A7 to A15 in the Appendix.

## Discussion

The present study had two objectives. First, this study tested for a potential linear increase in the proportion of AFAB children and adolescents attending a German gender identity service. Second, the possible presence of distinct clinical patterns related to the time youth first visited our clinic, sex assigned at birth, and clinical characteristics (psychological functioning and onset age) was explored.

Contrary to our hypothesis, the proportion of AFAB children and adolescents referred to the Hamburg GIS did not increase linearly over the ten years of evaluation. Throughout the study period, AFAB youth accounted for between 69% and 85% of the study sample, with fluctuations over the years but no clear linear trend. These findings contrast with those in the literature [1–3, 8, 10, 27–29]. In the second part of the study, however, we conducted an LCA and identified two clinical patterns based on the participants’ response patterns across multiple variables: an earlier cohort (with an average initial visit year in mid-2016) and a more recent cohort (with an average initial visit year around 2021). The proportion of AFAB individuals was significantly higher in the more recent cohort than in the earlier one. However, these results reflect differences between the identified cohorts rather than a modeled temporal trend, and thus do not allow for a conclusion on increasing numbers for AFAB youth, at least not in our center (in northern Germany).

The absence of a linear increase in the proportion of AFAB individuals in the Hamburg GIS, in contrast to the findings within other gender identity services (where an inversion of the sex ratio is frequently observed), can be attributed to a number of potential factors. First, many of the cited studies examined different time periods, emphasizing changes in referral numbers and sex ratios during the early 2000s and 2010s [1–3, 8]. The present study did not begin until 2013 and investigated more recent referrals up to 2023. Thus, it is possible that our study began after the reported shift in the sex ratio potentially occurred. This would also explain why most referrals were more AFAB throughout the entire study period, as was also the case in previous studies [16, 31–33].

Second, it is possible that there are ‘true’ national differences, as broader societal developments often unfold differently in different countries. These differences may be due to factors such as clinical or legal frameworks, cultural norms, levels of societal acceptance, and public discourse around gender diversity, all of which may influence the number and characteristics of children and adolescents who attend specialized gender identity services. Furthermore, national differences in treatment pathways, diagnostic procedures such as ‘eligibility criteria’, and available treatment options may influence these discrepancies.

Several explanations have been proposed for the overrepresentation of AFAB adolescents. First, AFAB individuals may experience less stigma when coming out as TGD compared to AMAB individuals, making it easier or more socially acceptable for them to transition and, consequently, to seek support from specialized gender identity services [2, 8]. Second, the earlier onset of puberty experienced by AFAB youth may result in earlier or more intense distress, leading to an earlier desire or pressure to seek help or to different gender identity trajectories among AFAB compared to AMAB individuals, who may present later in life, for example, in early adulthood [2]. Third, sociocultural norms and gender roles may make it easier or more socially validated for AFAB youth to explore masculine identities or come out as TGD than to identify as lesbian [27]. Notably, no single explanation has reached consensus, underscoring the need for an interdisciplinary perspective.

In addition to the sex differences, there were significant differences in terms of psychological functioning between the two latent classes. The more recent cohort experienced significantly fewer clinically meaningful mental health problems (measured with the CBCL total problems score) than the earlier cohort, which is consistent with recent Dutch and Norwegian studies [8, 10]. Societal acceptance of TGD identities may have increased in recent years (in Germany), resulting in fewer experiences of discrimination and consequently, less psychological distress. Given the increasing presence of TGD identities in public discourse and greater awareness of gender-affirming medical interventions, it may be that some young people are now accessing specialized services for reasons that differ from those of earlier cohorts, who may have sought support primarily due to clinical distress.

The post-hoc analyses indicated that the differences in terms of psychological functioning between the two latent classes varied somewhat when focusing on internalizing and externalizing problems . Specifically, individuals in the more recent cohort exhibited significantly fewer clinically meaningful externalizing problems but *more* internalizing problems than the earlier cohort, suggesting that improvements over time did not extend uniformly across all domains of psychological functioning. These results are only partly consistent with previous Dutch studies [8, 10], which reported decreases in externalizing problems over time and no temporal trend for internalizing problems. Furthermore, other studies relying on clinical diagnoses rather than self- or parent-report measures have reported increases in psychological functioning over time or no clear temporal trend [37, 38], highlighting the continued ambiguity in the existing evidence base.

Last but not least, the earlier and more recent cohorts significantly differed in terms of the onset age of GD. While most young people in the earlier cohort reported experiencing GD symptoms before puberty (pre-pubertal or late onset), those in the more recent cohort typically developed symptoms after puberty began (post-pubertal or early onset). These results may be considered consistent with the hypothesis proposed by several authors that the proportion of post-pubertal-onset cases has increased over time, particularly among AFAB adolescents [13, 16, 42], given the higher proportion of adolescents with a post-pubertal onset and those assigned female at birth in the more recent cohort compared to the earlier cohort. At first glance, the present findings may contradict the previous results of our previous study authored by Fahrenkrug et al. [16], who reported a significant linear association between a post-pubertal onset age and higher levels of internalizing problems. However, these differing results may reflect the distinct methodological approaches employed. While in our previous study [16], we focused on internalizing problems, the current study focused on total problems. Interestingly, we found similar results to Fahrenkrug et al. when looking at the internalizing problems in the more recent vs. earlier cohorts. Another important difference is that the current study used the parent-report, whereas Fahrenkrug et al. [16] used the self-report form of the CBCL (Youth Self-Report). Our current findings therefore support the hypothesis proposed by several authors [13, 16, 42] that there may be an increasing number of adolescents who experience a post-pubertal onset of GD, emphasizing the need for long-term research to inform evidence-based care for current referrals.

The COVID-19 pandemic may have impacted the findings, given that the earlier cohort’s average initial visit took place before the pandemic, whereas the more recent cohort’s average initial visit occurred afterward. For instance, increased time spent online, greater exposure to transgender-related content on social media, greater visibility and acceptance of gender diversity, and changes in access to mental healthcare services during this period may have contributed to changes in gender identity exploration and in help-seeking behavior among TGD youth. Notably, increases in internalizing mental health problems were also observed among (cisgender) youth in the broader general population during the pandemic, which may further account for cohort differences [57]. These contextual factors should be considered when interpreting the results.

In summary, the results of the present study suggest the presence of distinct clinical cohorts of children and adolescents attending a specialized gender identity service in Germany. Current treatment protocols for gender-affirming medical interventions in Europe are based on the *Dutch Protocol* and its associated outcome studies [18, 58–61], which applied relatively strict eligibility criteria from the early 2000, such as low levels of co-occurring psychological functioning and a pre-pubertal onset of GD. As a result, adolescents with post-pubertal onset were not included in the original outcome studies. Thus, more long-term research is needed to determine the most suitable care approaches for recent cohorts of TGD adolescents, ensuring they receive the best possible treatment.

The present study has several notable strengths. To the best of our knowledge, it is the first to investigate a potential linear increase in the proportion of AFAB referrals and to identify clinical patterns among children and adolescents attending a specialized gender identity service in Germany. We analyzed a large clinical sample and employed advanced statistical analyses, including LCA, to identify potential clinical patterns.

However, several limitations should also be acknowledged. First, the classes or cohorts identified through LCA are statistical constructs rather than diagnostically defined groups or naturally occurring groups. They lack a theoretical basis, and their existence depends on the specific sample and variables included in the analysis. Consequently, the findings are probably not generalizable. Replication in other samples and settings is needed to validate these results.

Second, although evaluating changes in referral numbers was not a primary aim of the study, it would have been valuable to do so, as has been done in other studies. However, population-based representative data were not available. Referral numbers from the Hamburg GIS do not reflect actual demand, as they have been affected by limited and fluctuating personnel over time, influencing the number of first presentations. Additionally, the location of the center (Hamburg, largest city in northern Germany, but not representative for the whole of Germany) and growing waiting lists further restricted representativeness. Figure A2 in the Appendix provides an overview of referral numbers, which should be interpreted with caution.

Third, we opted to use a parent-report measure (the CBCL) across all age groups. Although using a self-report instrument (such as the Youth-Self Report) would have been feasible and informative for adolescents, we prioritized using a single informant to ensure methodological consistency and comparability of outcomes across age groups. Additionally, the CBCL version from 1991 was used throughout the entire study period, although the measure has since been updated. Consequently, some items and the associated norm scores may no longer fully reflect current populations.

## Conclusions

The present study examined a potential linear increase in the proportion of AFAB referrals over time and the presence of distinct clinical patterns among children and adolescents attending a gender identity service for youth in northern Germany. Although no linear trend was observed in the sex ratio over time, exploratory analyses revealed notable differences in various demographic and clinical features between an earlier and more recent cohort of clinically referred youth with GD, as identified in the LCA. More long-term evaluation studies are needed to assess the outcomes of different kinds of gender-affirming care for more recent cohorts of TGD youth.

.

## Supplementary Information

Below is the link to the electronic supplementary material.

Supplementary Material 1

## Data Availability

No datasets were generated or analysed during the current study.
