# Supplementary material for: Ten years of a gender identity service for children and adolescents in Germany: demographic and clinical patterns
Source: Eur Child Adolesc Psychiatry. 2026 Jun 8;35(10):3157–68. doi: 10.1007/s00787-026-03080-w (PMC13696553; doi:10.1007/s00787-026-03080-w)
Supplement: Supplementary file 1 [file 787_2026_3080_MOESM1_ESM.docx]

**APPENDIX**

| **Table A1.** Demographic characteristics in the referral sample (*N* = 1,413). | | |
| --- | --- | --- |
|  | *n* | % |
| **Sex assigned at birth** |  |  |
| Assigned male at birth | 350 | 24.8 |
| Assigned female at birth | 1,063 | 75.2 |
| **Age group** |  |  |
| Children (up to 10 years old) | 150 | 10.6 |
| Adolescents (11 years or older) | 1,263 | 89.4 |
|  | *M* | *SD* |
| **Age at assessment** (in years) | 14.75 | 2.89 |
|  | | |

| **Table A2.** Exploratory multiple logistic regression analysis in the sample of all referrals (*N* = 1,413): Association between year of initial visit (centered around the year 2013) and sex assigned at birth (0 = male, 1 = female). | | | | | |
| --- | --- | --- | --- | --- | --- |
|  | *B* | *SE* | OR | 95% CI for OR | *p* |
| Intercept | −0.43 | 0.20 | 0.65 |  | .034 |
| Age group (0 = child, 1 = adolescent) | 1.69 | 0.18 | 5.40 | [3.80, 7.68] | <.001 |
| Year of initial visit (centered) | 0.02 | 0.02 | 1.02 | [0.97, 1.06] | .495 |
| Results of the final model: *χ*^2^(2) = 89.38, *p* < .001, *R^2^* (Nagelkerke) = .09 | | | | | |

**Figure A1:** Total number of referrals (*N* = 1,413) to the Hamburg Gender Identity Service for children and adolescents (Hamburg GIS) between 2013 and 2023.

| **Table A3.** Internalizing and externalizing problems as well as total problem score based on cross-sex norms (instead of norms based on sex assigned at birth) in the study population (*n* = 779). | | |
| --- | --- | --- |
|  | *n* | % |
| **CBCL internalizing scale** |  |  |
| Non-clinical range (*T* scores ≤ 63) | 362 | 46.5 |
| Clinical range (> 90th percentile; *T* scores > 63) | 417 | 53.5 |
| **CBCL externalizing scale** |  |  |
| Non-clinical range (*T* scores ≤ 63) | 634 | 81.4 |
| Clinical range (> 90th percentile; *T* scores > 63) | 145 | 18.6 |
| **CBCL total problems** (based on cross-sex norms) |  |  |
| Non-clinical range (*T* scores ≤ 63) | 472 | 60.6 |
| Clinical range (> 90th percentile; *T* scores > 63) | 307 | 39.4 |
| CBCL, Child Behavior Checklist. | | |

| **Table A4.** Additional exploratory analyses for the association between year of initial visit and sex assigned at birth with internalizing problems as predictor (study population: *n* = 779). | | | | | |
| --- | --- | --- | --- | --- | --- |
|  | *B* | *SE* | OR | 95% CI for OR | *p* |
| Intercept | −70.77 | 72.78 | 0.00 |  | .331 |
| Year of initial visit (centered) | 0.04 | 0.04 | 1.04 | [0.97, 1.11] | .333 |
| Age group (0 = child, 1 = adolescent) | 2.04 | 0.27 | 7.65 | [4.54, 12.90] | <.001 |
| CBCL clinical range for internalizing problems (0 = non-clinical, 1 = clinical) | 0.10 | 0.19 | 1.10 | [0.76, 1.61] | .610 |
| Post-pubertal onset of GD (= 1, pre-pubertal = 0) | −0.12 | 0.23 | 0.89 | [0.57, 1.39] | .608 |
| No GD or missing information (= 1, pre-pubertal = 0) | −0.46 | 0.31 | 0.63 | [0.35, 1.15] | .136 |
| Multiple logistic regression analysis, year of assessment centered on the year 2013, model fit: *χ*^2^(5) = 78.07, *p* < .001, *R^2^* (Nagelkerke) = .15  GD, gender dysphoria. | | | | | |

| **Table A5.** Additional exploratory analyses for the association between year of initial visit and sex assigned at birth with externalizing problems as predictor (study population: *n* = 779). | | | | | |
| --- | --- | --- | --- | --- | --- |
|  | *B* | *SE* | OR | 95% CI for OR | *p* |
| Intercept | −77.88 | 73.20 | 0.00 |  | .287 |
| Year of initial visit (centered) | 0.04 | 0.04 | 1.04 | [0.97, 1.12] | .290 |
| Age group (0 = child, 1 = adolescent) | 2.10 | 0.27 | 8.14 | [4.83, 13.71] | <.001 |
| CBCL clinical range for externalizing problems (0 = non-clinical, 1 = clinical) | 0.53 | 0.26 | 1.69 | [1.01, 2.83] | .045 |
| Post-pubertal onset of GD (= 1, pre-pubertal = 0) | −0.07 | 0.23 | 0.93 | [0.60, 1.45] | .750 |
| No GD or missing information (= 1, pre-pubertal = 0) | −0.48 | 0.31 | 0.62 | [0.34, 1.13] | .118 |
| Multiple logistic regression analysis, year of assessment centered on the year 2013, model fit: *χ*^2^(5) = 82.11, *p* < .001, *R^2^* (Nagelkerke) = .16  GD, gender dysphoria. | | | | | |

| **Table A6.** Additional exploratory analyses for the association between year of initial visit and sex assigned at birth, with total problems based on cross-sex norms (instead of birth-assigned sex) as predictor (study population: *n* = 779). | | | | | |
| --- | --- | --- | --- | --- | --- |
|  | *B* | *SE* | OR | 95% CI for OR | *p* |
| Intercept | −80.49 | 73.09 | 0.00 |  | .271 |
| Year of initial visit (centered) | 0.04 | 0.04 | 1.04 | [0.97, 1.12] | .274 |
| Age group (0 = child, 1 = adolescent) | 2.06 | 0.26 | 7.82 | [4.66, 13.11] | <.001 |
| CBCL clinical range for total problems based on cross-sex norms (0 = non-clinical, 1 = clinical) | 0.40 | 0.20 | 1.49 | [1.01, 2.20] | .044 |
| Post-pubertal onset of GD (= 1, pre-pubertal = 0) | −0.12 | 0.22 | 0.88 | [0.57, 1.37] | .583 |
| No GD or missing information (= 1, pre-pubertal = 0) | −0.51 | 0.31 | 0.60 | [0.33, 1.10] | .096 |
| Multiple logistic regression analysis, year of assessment centered on the year 2013, model fit: *χ*^2^(5) = 81.95, *p* < .001, *R^2^* (Nagelkerke) = .15  GD, gender dysphoria. | | | | | |

| **Table A7.** Model fit for additional exploratory analyses of latent classes with internalizing problems instead of the total problem score as categorical indicator. | | | | | | | |
| --- | --- | --- | --- | --- | --- | --- | --- |
| Classes | LL | BIC | aBIC | Npar | Entropy | LMR-LRT | *p* |
| **2 (vs. 1)** | **-3480.58** | **7041.05** | **7002.94** | **12** | **.71** | **152.35** | **< .001** |
| 3 (vs. 2) | -3463.13 | 7046.11 | 6988.95 | 18 | .71 | 34.03 | 0.062 |
| 4 (vs. 3) | -3449.48 | 7058.78 | 6982.55 | 24 | .72 | 26.63 | 0.176 |
| (a)BIC, (sample-size adjusted) Bayesian Information Criterion; LL, Loglikelihood H0 value; LMR-LRT, Lo-Mendell-Rubin adjusted likelihood ratio test; Npar, number of free parameters. | | | | | | | |

| **Table A8.** Average latent class probabilities for most likely latent class membership (row) by latent class (column) in additional exploratory analyses with internalizing problems instead of the total problem score as categorical indicator. | | |
| --- | --- | --- |
|  | 1 | 2 |
| 1 | **.931** | .069 |
| 2 | .097 | **.932** |

| **Table A9.** Conditional response probabilities for categorical indicators and means (standard errors) for continuous indicators by latent class in additional exploratory analyses with internalizing problems instead of the total problem score as categorical indicator. | | | | |
| --- | --- | --- | --- | --- |
|  | **Class 1: more recent cohort**  (*n* = 368, 47%) | **Class 2: earlier cohort**  (*n* = 410, 53%) | **OR** | ***p*** |
| **Sex assigned at birth** |  |  |  |  |
| Assigned male at birth | .19 | .24 |  |  |
| Assigned female at birth | .82 | .76 | 1.40 | < .001 |
| **CBCL internalizing scale** |  |  |  |  |
| Non-clinical range (*T* scores ≤ 63) | .44 | .48 |  |  |
| Clinical range (*T* scores > 63) | .56 | .52 | 1.18 | <.001 |
| **Onset age** |  |  |  |  |
| Pre-pubertal onset of GD | .29 | **.64** |  |  |
| Post-pubertal onset of GD | **.62** | .26 | 4.28 | < .001 |
| No GD diagnosis or missing information | .09 | .10 | 0.89 | .001 |
| Year of assessment | 2020.89 (0.13) | 2016.53 (0.11) |  | <.001 |
| CBCL, Child Behavior Checklist; GD, gender dysphoria. | | | | |

| **Table A10.** Model fit for additional exploratory analyses of latent classes with externalizing problems instead of the total problem score as categorical indicator. | | | | | | | |
| --- | --- | --- | --- | --- | --- | --- | --- |
| Classes | LL | BIC | aBIC | Npar | Entropy | LMR-LRT | *p* |
| **2 (vs. 1)** | **-3314.85** | **6709.59** | **6671.48** | **12** | **.71** | **156.40** | **< .001** |
| 3 (vs. 2) | -3302.56 | 6725.15 | 6667.99 | 18 | .65 | 23.80 | 0.010 |
| 4 (vs. 3) | -3283.52 | 6726.84 | 6650.63 | 24 | .71 | 37.32 | 0.155 |
| (a)BIC, (sample-size adjusted) Bayesian Information Criterion; LL, Loglikelihood H0 value; LMR-LRT, Lo-Mendell-Rubin adjusted likelihood ratio test; Npar, number of free parameters. | | | | | | | |

| **Table A11.** Average latent class probabilities for most likely latent class membership (row) by latent class (column) in additional exploratory analyses with externalizing problems instead of the total problem score as categorical indicator. | | |
| --- | --- | --- |
|  | 1 | 2 |
| 1 | **.900** | .100 |
| 2 | .066 | **.934** |

| **Table A12.** Conditional response probabilities for categorical indicators and means (standard errors) for continuous indicators by latent class in additional exploratory analyses with externalizing problems instead of the total problem score as categorical indicator. | | | | |
| --- | --- | --- | --- | --- |
|  | **Class 1: earlier cohort**  (*n* = 409, 52%) | **Class 2: more recent cohort**  (*n* = 370, 48%) | **OR** | ***p*** |
| **Sex assigned at birth** |  |  |  |  |
| Assigned male at birth | .24 | .19 |  |  |
| Assigned female at birth | .76 | .82 | 0.72 | < .001 |
| **CBCL externalizing scale** |  |  |  |  |
| Non-clinical range (*T* scores ≤ 63) | .78 | .85 |  |  |
| Clinical range (*T* scores > 63) | .22 | .15 | 1.63 | <.001 |
| **Onset age** |  |  |  |  |
| Pre-pubertal onset of GD | **.64** | .29 |  |  |
| Post-pubertal onset of GD | .26 | **.62** | 0.23 | < .001 |
| No GD diagnosis or missing information | .10 | .09 | 1.20 | .001 |
| Year of assessment | 2016.52 (0.11) | 2020.87 (0.14) |  | <.001 |
| CBCL, Child Behavior Checklist; GD, gender dysphoria. | | | | |

| **Table A13.** Model fit for additional exploratory analyses of latent classes with total problem score based on cross-sex norms (instead of norms based on sex assigned at birth) as categorical indicator. | | | | | | | |
| --- | --- | --- | --- | --- | --- | --- | --- |
| Classes | LL | BIC | aBIC | Npar | Entropy | LMR-LRT | *p* |
| **2 (vs. 1)** | **-3464.94** | **7009.77** | **6971.66** | **12** | **.71** | **152.30** | **< .001** |
| 3 (vs. 2) | -3451.84 | 7023.52 | 6966.36 | 18 | .65 | 25.56 | 0.149 |
| 4 (vs. 3) | -3433.58 | 7026.95 | 6950.74 | 24 | .72 | 35.63 | 0.052 |
| (a)BIC, (sample-size adjusted) Bayesian Information Criterion; LL, Loglikelihood H0 value; LMR-LRT, Lo-Mendell-Rubin adjusted likelihood ratio test; Npar, number of free parameters. | | | | | | | |

| **Table A14.** Average latent class probabilities for most likely latent class membership (row) by latent class (column) in additional exploratory analyses with total problem score based on cross-sex norms (instead of norms based on sex assigned at birth) as categorical indicator. | | |
| --- | --- | --- |
|  | 1 | 2 |
| 1 | **.901** | .099 |
| 2 | .067 | **.933** |

| **Table A15.** Conditional response probabilities for categorical indicators and means (standard errors) for continuous indicators by latent class in additional exploratory analyses with total problem score based on cross-sex norms (instead of norms based on sex assigned at birth) as categorical indicator. | | | | |
| --- | --- | --- | --- | --- |
|  | **Class 1: earlier cohort**  (*n* = 409, 53%) | **Class 2: more recent cohort**  (*n* = 370, 47%) | **OR** | ***p*** |
| **Sex assigned at birth** |  |  |  |  |
| Assigned male at birth | .24 | .19 |  |  |
| Assigned female at birth | .76 | .81 | 0.72 | < .001 |
| **CBCL total problems score** |  |  |  |  |
| Non-clinical range (*T* scores ≤ 63) | .59 | .63 |  |  |
| Clinical range (*T* scores > 63) | .41 | .37 | 1.18 | <.001 |
| **Onset age** |  |  |  |  |
| Pre-pubertal onset of GD | **.64** | .29 |  |  |
| Post-pubertal onset of GD | .26 | **.62** | 0.24 | < .001 |
| No GD diagnosis or missing information | .10 | .09 | 1.19 | .001 |
| Year of assessment | 2016.52 (0.11) | 2020.88 (0.13) |  | <.001 |
| CBCL, Child Behavior Checklist; GD, gender dysphoria. | | | | |
